# Improving Advanced Care Planning Discussions at an Internal Medicine Clinic

**DOI:** 10.7759/cureus.75156

**Published:** 2024-12-05

**Authors:** Michael J Brockman, Jared J Bies, Hedaia Algheriani, John P Aparece, Marco Cazares Parson, Raul Del Toro Mijares, Eyoab Massebo, Ramon G Valles, Hein Zay, Abhizith Deoker, Lisa A Hechanova, Brian P Edwards, Irene Sarosiek

**Affiliations:** 1 Internal Medicine, Texas Tech University Health Sciences Center El Paso Paul L. Foster School of Medicine, El Paso, USA; 2 Internal Medicine, Texas Tech University Health Sciences Center El Paso, El Paso, USA; 3 Internal Medicine/Nephrology, Texas Tech University Health Sciences Center El Paso, El Paso, USA

**Keywords:** advance care planning, general internal medicine, palliative care education, role of palliative care, supportive and palliative care

## Abstract

Objective: The project aimed to standardize advanced care planning (ACP) at an internal medicine clinic by initiating physician-patient communication regarding the patient’s knowledge, understanding, and openness to pursuing advanced medical directives.

Methods: Data collection was conducted from February 1 to April 1, 2024, with the study concluding on April 24, 2024. ACP was facilitated through an initial standardized six-question pre-intervention survey in both English and Spanish. This pre-survey included questions on prior survey exposure within the past three months, current age, existing or previous medical conditions, possession of an advance directive (e.g., living will or durable power of attorney for healthcare), and interest in learning more about advanced medical directives. For patients interested in learning more, standardized educational materials from the National Institute on Aging were provided, along with a Texas out-of-hospital do-not-resuscitate (OOH-DNR) order, a Medical Power of Attorney form, and instructions in both English and Spanish. Post-education, patients completed a post-intervention survey asking if they had previously discussed advanced medical directives with a physician. The survey also included Likert scale questions about the discussion’s usefulness, comfort with end-of-life discussions, perceived importance of advanced directives, and likelihood of completing an advance directive.

Results: During the three months, 52 patients completed the pre-intervention survey, with an average age of 59 years. Hypertension, dyslipidemia, and diabetes were the most common conditions among participants. Statistical tests indicated no significant difference between patients’ age or number of comorbidities and possession of an advance directive (p > 0.05), nor was there a significant association between these variables and interest in learning more about advanced directives (p > 0.05). However, post-intervention survey results showed a significant correlation between age and prior discussions about advanced directives (p = 0.013) and between the number of comorbidities and having had past discussions (p = 0.025). Only 1.2% of patients reported having advanced directives before this study, highlighting a substantial gap in documentation.

Conclusion: This project revealed a notable gap in ACP documentation among patients at the internal medicine clinic, with very few patients having advanced directives prior to the intervention. While age and comorbidity count were not significantly associated with interest in advanced directives, older patients and those with more comorbidities were more likely to have had previous discussions. This underscores the need for targeted efforts to encourage ACP, particularly among younger patients and those with fewer medical conditions. Standardized educational resources effectively facilitated discussions, raising awareness and promoting engagement in ACP.

## Introduction

The internal medicine clinic provides primary care to a diverse population, serving residents of neighboring nations near the border. Effective record-keeping is critical in this dynamic community to ensure continuity of care and facilitate decision-making during emergencies. Despite the importance of advanced care planning (ACP) and advanced medical directives in supporting informed decision-making, a meta-analysis from 2011 to 2016 found that only 36.7% of patients, and 38.2% of those with chronic illnesses, had documented directives [1]. The number of patients with such documentation at this clinic is unknown, and there is no standardized method to educate patients on ACP. This gap highlights the need for early physician-patient discussions and structured resources to improve patient care and preparedness for future healthcare decisions.

## Materials and methods

This study was conducted at the internal medicine clinic and aimed to address ACP and advanced medical directives with patients to improve overall patient care. The objective was to initiate discussions about ACP and provide advance medical directive instructions and resources to all willing patients. This aimed to enhance awareness, education, and the prevalence of advanced medical directives among clinic patients. The study included all patients aged 18 years and older with appointments at the clinic from February 1 to April 1, 2024. Patients under 17 years, those who refused to participate, those who did not complete the post-intervention survey before discharge, and those who answered “No” to Question 6 of the pre-appointment survey were excluded from the study. This single-group, cross-sectional study was conducted without patient randomization, with participants assigned to the study group based on inclusion and exclusion criteria.

Resident physicians conducted the study within the internal medicine residency program, and institutional board review (IRB) exemption was granted on January 31, 2024 (E24038), after initial submission on December 6, 2023, and necessary modifications. Data collection took place from February 1 to April 1, 2024. Data collected included the patient's age, comorbidities, advance medical directive completion status, and responses to a post-intervention survey when applicable. A numerical code was assigned to each patient, recorded separately in a Coded Identifier List to link pre-appointment and post-intervention surveys, without containing identifying information. The Excel (Microsoft Corp., Redmond, Washington, DC) file containing the data was saved on a secured, password-protected computer in the principal investigator's locked office. All personnel received training on proper data handling to minimize privacy risks, and data were stored on an encrypted device. Additionally, paper documents were destroyed after being digitized.

Data confidentiality was maintained by recording information in a Microsoft Excel document, with columns representing variables and each row representing individual participants. No identifiable information, such as names or biographical data, was recorded. Each patient received a random numerical code to link surveys, ensuring anonymity. The Excel document was stored on a secure device accessible only to IRB-approved personnel. Paper surveys were kept in the principal investigator’s (PI) locked office, and all documents identifying subjects were destroyed upon study completion per IRB procedures.

During the study, all eligible patients who agreed to participate received an anonymous pre-intervention survey in English or Spanish at intake to capture baseline information on ACP. This survey assessed whether the patient had an advance medical directive and whether they wanted to receive education on ACP (Appendix A). Patients had three to four minutes to complete this survey to establish baseline knowledge of ACP and determine if they had completed an advance medical directive. Patients could opt out of the study at any time. For those interested in additional information on ACP, internal medicine residents provided standardized education from the National Institute on Aging website. This educational material explained ACP, the nature and importance of advanced medical directives, and the process for completing them. Patients had five to 10 minutes to review the information during the visit.

Patients who expressed interest in learning about ACP received printed copies of an out-of-hospital do-not-resuscitate (OOH-DNR) order and a Medical Power of Attorney form, along with bilingual instructions for form completion (Appendices B, C). Following the educational intervention, patients completed a post-intervention survey in English or Spanish, providing feedback on the education and their opinions on end-of-life planning (Appendix D). This survey, completed within three to four minutes, allowed patients to express their views on the education provided, maintaining anonymity and excluding any identifying characteristics.

## Results

**Figure 1:**
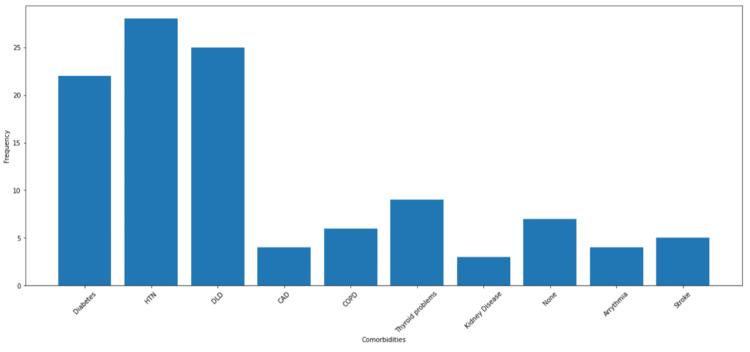
Distribution of diagnoses among patients HTN: Hypertension; DLD: Dyslipidemia; CAD: Coronary Artery Disease; COPD: Chronic Obstructive Pulmonary Disease

During the three months, 52 patients completed the ACP pre-intervention survey. The average age of the responding patients was found to be 59 years. The survey revealed that the three most prevalent diseases were hypertension (n=28, 53.8%), dyslipidemia (n=28, 48.1%), and diabetes (n=22, 42.3%). These results, along with the other surveyed comorbidities, are represented in Figure 1.

**Figure 2:**
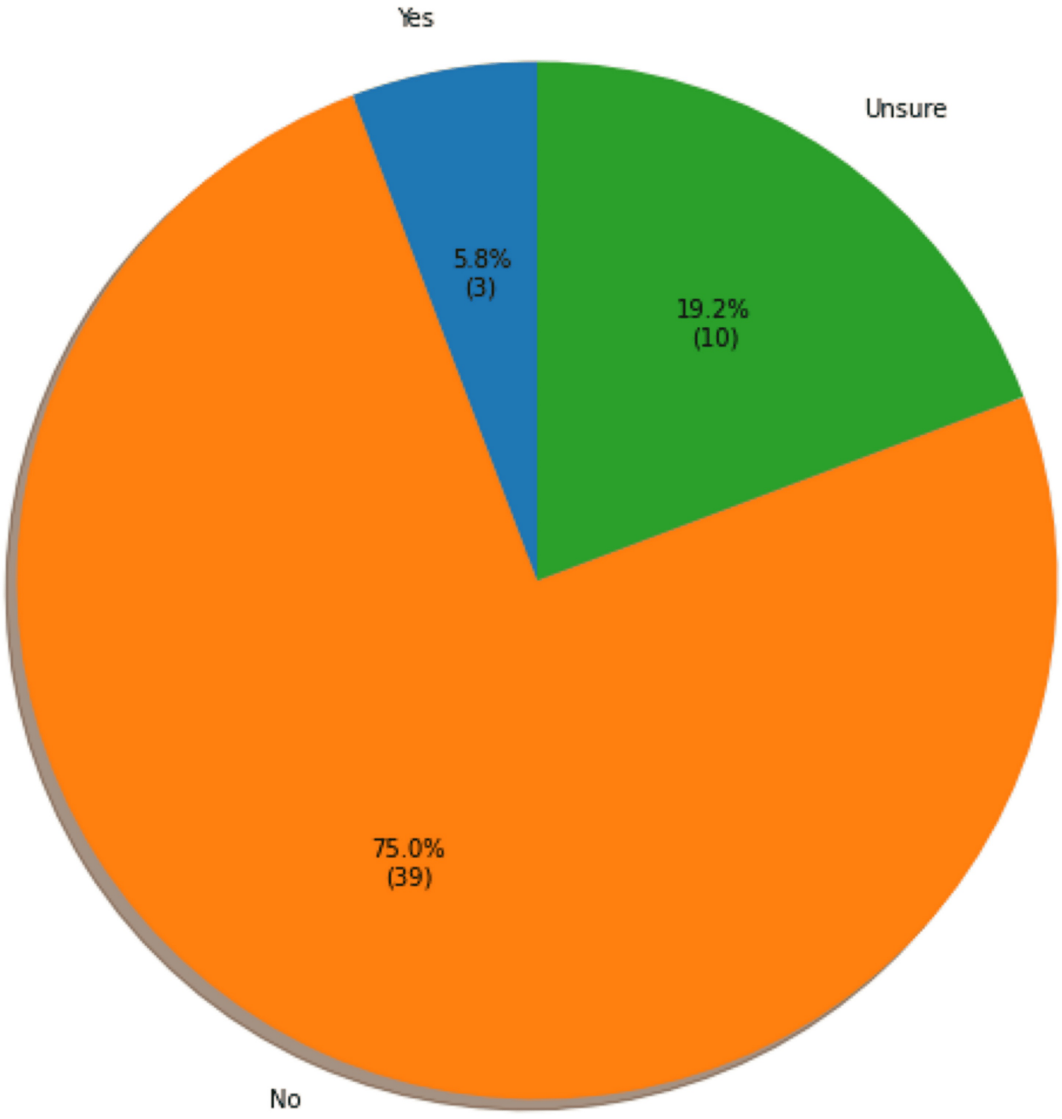
Patient responses on having advanced medical directives (N=52)

The fourth question of the ACP pre-intervention survey asked if patients had advanced medical directives prior to the visit. Of the 52 responding patients, three reported having advanced directives (1.2%), 10 were unsure if they had advanced directives (19.2%), and 39 reported not having advanced directives (75%) (Figure 2). A t-test was performed and found no statistically significant difference between patients' age or number of comorbidities and having an advance medical directive (p > 0.05).

**Figure 3:**
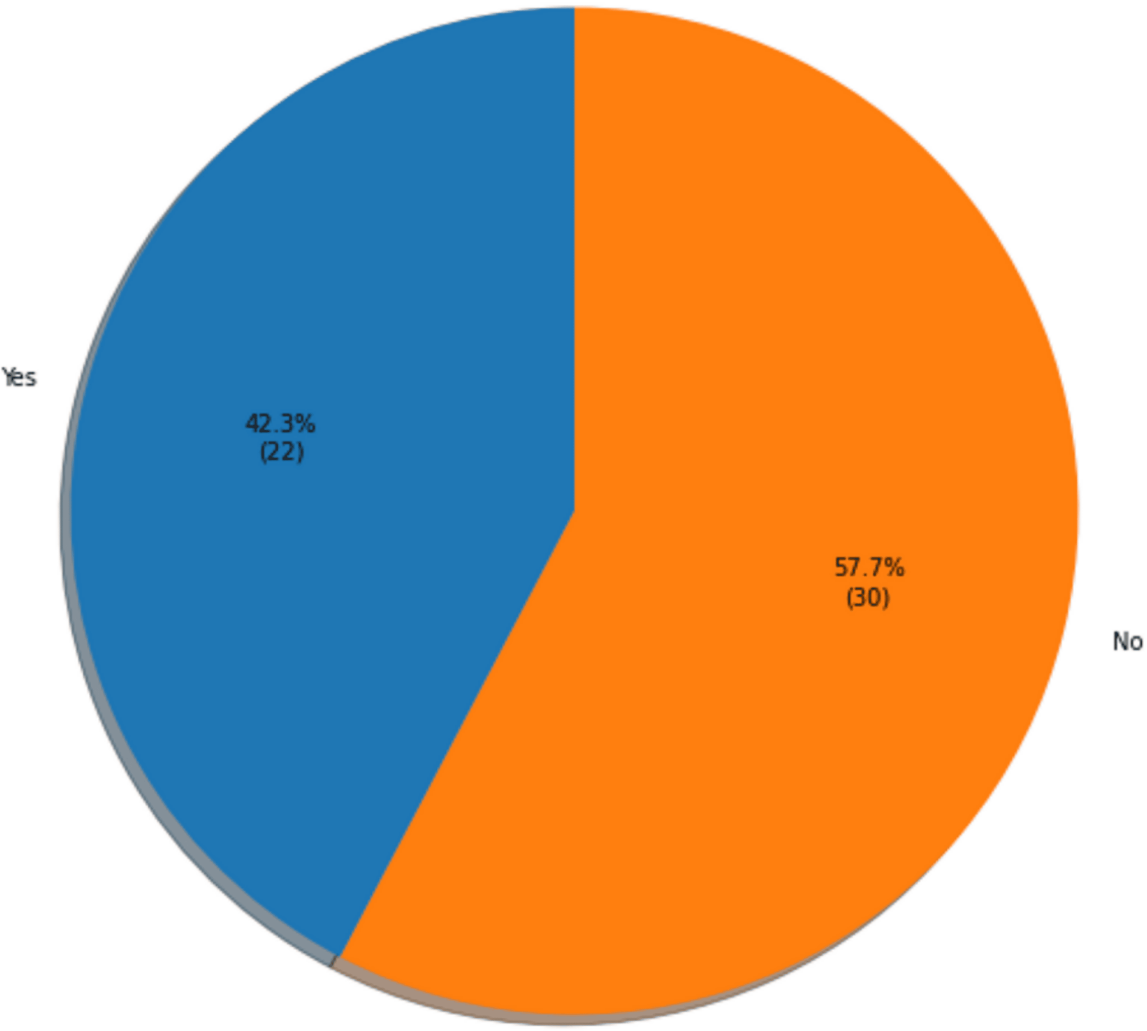
Patient interest in learning about advanced medical directives (N=52)

The ACP pre-intervention survey also captured patients' attitudes toward learning more about advanced medical directives during their appointments. Twenty-two patients expressed interest in learning more about advanced medical directives, while 30 declined (Figure 3). The performed t-test suggested no statistically significant difference between patients' number of comorbidities or age and their interest in learning more about advanced medical directives (p > 0.05).

**Figure 4:**
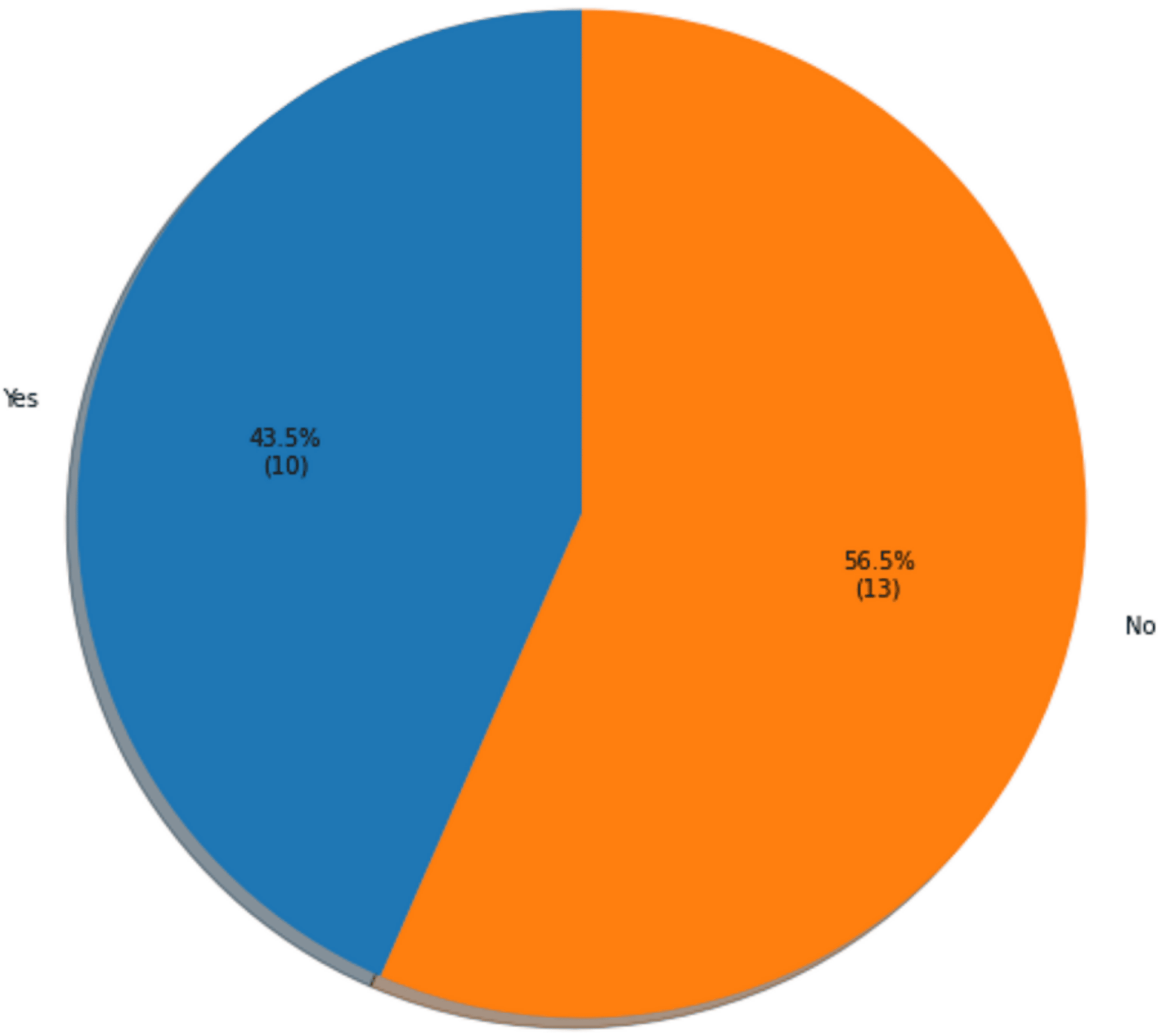
Previous discussions about advanced medical directives with a provider (N=23)

Of the 52 patients who completed the ACP pre-intervention survey, 23 completed the ACP post-intervention survey. The initial question in the survey asked if patients had ever had discussions about advanced medical directives with a physician prior to the visit. Ten patients (43.5%) reported having such discussions in the past, while 13 (56.5%) stated they had never had advance medical directive discussions before (Figure 4). The performed t-test suggested a statistical significance between the patient's age and the likelihood of having had an advanced medical directives discussion prior to this study (p = 0.013). There was also a significant correlation between the number of comorbidities and having had past discussions (p = 0.025).

**Figure 5:**
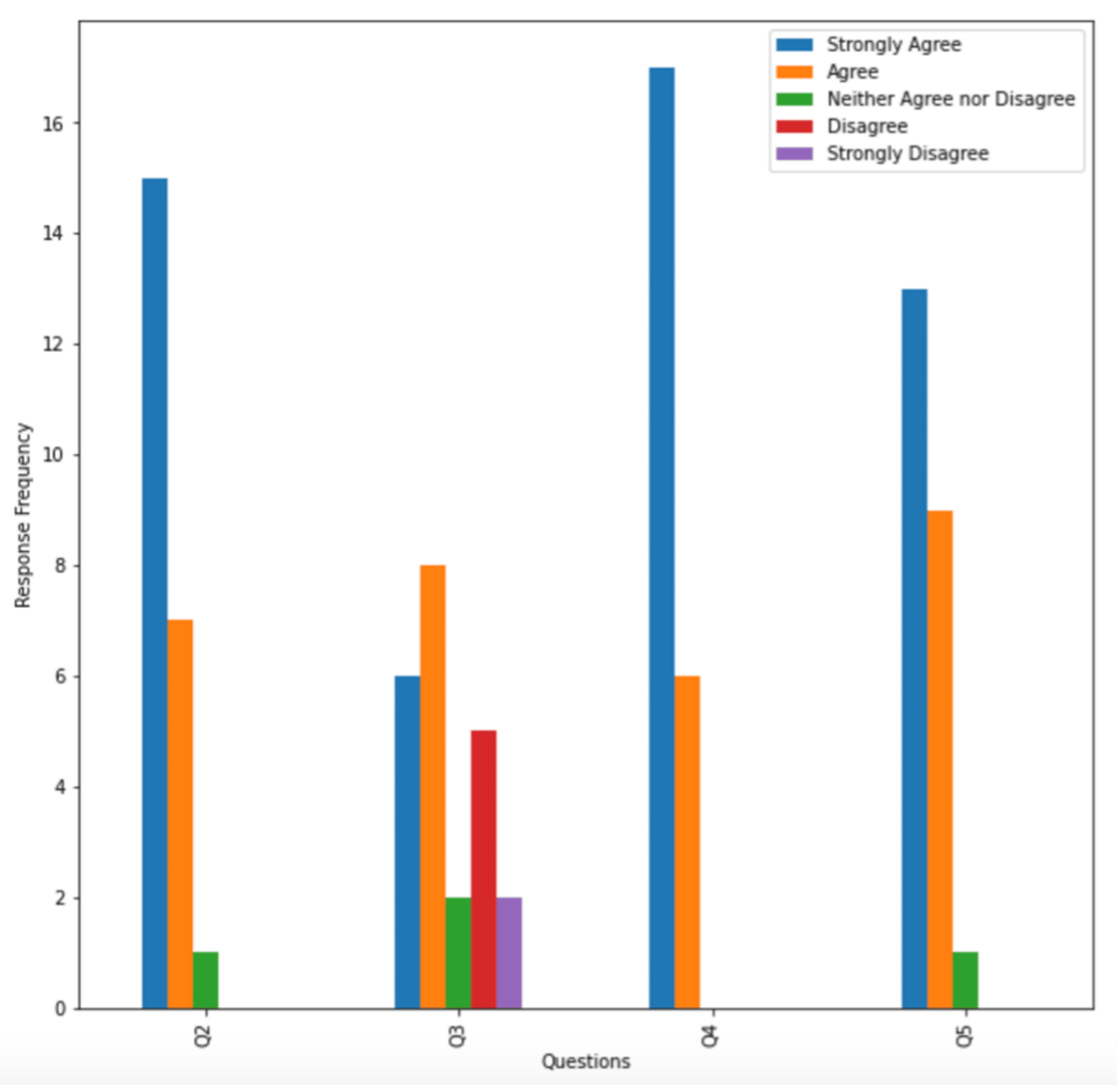
Patient responses to Likert-scale questions on advanced medical directives

Questions 2 through 5 were Likert scale questions. Question 2, “This advance medical directive conversation was helpful,” had 15 patients (65.2%) respond “strongly agree,” seven (30.4%) respond “agree,” and one (4.3%) respond “neither agree nor disagree.” Question 3, “I am uncomfortable with end-of-life discussions," had six patients (26.1%) respond “strongly agree,” eight (34.8%) respond “agree,” two (8.7%) respond “neither agree nor disagree,” five (21.7%) respond “disagree,” and two (8.7%) respond “strongly disagree.” Question 4, “I see the importance of having advanced medical directives,” had 17 patients (73.9%) respond “strongly agree” and six (26.1%) respond “agree.” Question 5, “I now plan on completing my advanced medical directives in the future,” had 13 patients (56.5%) respond “strongly agree,” nine (39.1%) respond “agree,” and one (4.3%) respond “neither agree nor disagree.” These results are represented in Figure 5. As for the reasons for not pursuing advanced directives, three patients (13.0%) responded that they did not know how to start, and one patient (4.3%) stated they did not find it necessary. The remaining patients did not respond.

## Discussion

Publishing these findings is extremely significant for several reasons. Firstly, by standardizing ACP discussions, this study demonstrates a clear pathway to improving patient care through better preparation for end-of-life decisions [1]. Ensuring that patients' wishes are known and respected can reduce stress for both patients and their families during critical times, particularly when patients are unable to communicate their preferences [1]. This aligns with prior research by Silveira et al., which highlighted that patients with advanced directives are more likely to receive care consistent with their values and wishes, reducing the burden on surrogate decision-makers [1].

This intervention also provided valuable education to patients, many of whom may not have been previously aware of or understood advanced directives [2]. Education is a critical component in ACP, as demonstrated by Bravo et al., who found that structured educational interventions significantly increased the completion rates of advanced directives among community-dwelling older adults [3]. The inclusion of culturally and linguistically appropriate materials, as implemented in this study, is crucial for ensuring that diverse patient populations can fully engage in ACP discussions [4]. Sudore et al. emphasized the need for culturally sensitive ACP discussions, particularly in diverse populations similar to those served in this study [4].

Moreover, the project’s methodology and findings can serve as a model for other clinics and healthcare institutions [5]. A systematic approach to ACP discussions promotes the integration of standardized practices into routine care, leading to widespread improvements in how advanced directives are handled across different healthcare settings [5]. Studies by Morrison et al. and Detering et al. have further supported that culturally tailored ACP interventions lead to higher engagement and completion rates among minority groups [6,7].

This is particularly important in primary care settings, where long-term patient-provider relationships can facilitate ongoing ACP discussions, as suggested by Tung et al. [8]. The standardized approach used in this study is in line with best practices outlined by Lund et al., who found that systematic implementation of ACP in clinical settings can lead to higher rates of advance directive completion and greater patient satisfaction [2]. Similar findings were reported by Jimenez et al., who highlighted the global lessons learned from systematic reviews of ACP [9]. Rietjens et al. also emphasized the importance of continuity of care in successful ACP implementation [10].

The results of this study further highlight its significance to current literature on ACP [9]. During the three months, 52 patients completed the pre-intervention survey, with an average age of 59 years [1]. The survey revealed that hypertension, dyslipidemia, and diabetes were the most prevalent diseases among participants, conditions often associated with higher healthcare utilization and potential complications that necessitate clear advance care plans [1]. Notably, only 1.2% of patients reported having advanced directives prior to the study, underscoring a significant gap in ACP documentation in this population [1]. This finding is consistent with national data reported by Yadav et al., which showed that approximately one-third of U.S. adults have completed some form of advance directive [11]. Furthermore, a study by Rao et al. indicated that chronic conditions like those prevalent in this population often correlate with a higher desire for ACP, yet the completion rates remain low due to systemic barriers [12].

The post-intervention survey results showed a substantial increase in the number of patients who had discussed advanced directives, with 43.5% of participants engaging in these conversations [1]. A significant number of patients found the discussions helpful and recognized the importance of having advanced directives, which is supported by evidence from Jimenez et al., who demonstrated that ACP discussions improve patient and family satisfaction with end-of-life care [9]. These results align with findings by Wright et al., who demonstrated that early ACP discussions significantly reduced anxiety and depression among patients with serious illnesses [13]. Similarly, a randomized controlled trial by Houben et al. showed that ACP discussions are associated with better alignment of care with patients' wishes and fewer unwanted hospitalizations near the end of life [5].

Furthermore, the study addresses significant gaps in the literature regarding ACP in specific demographics, particularly older adults with multiple comorbidities [8]. Research by Tung et al. suggests that older adults with multiple comorbidities are more likely to engage in ACP discussions, a finding that was corroborated by this study's data, which highlighted the association between comorbidities and prior ACP discussions [8]. Additionally, studies such as those by Song et al. have shown that tailored interventions, like the ones implemented in this project, can significantly increase the completion rates of advanced directives among patients, particularly in populations with chronic illnesses [14]. Zhang et al. also noted that patients with multiple chronic conditions benefit most from ACP, as it helps align their complex care needs with their values [15].

The implications of these findings are broad, extending beyond individual patient care to inform policy and training programs for healthcare providers [5]. Integrating ACP discussions early in the patient care process, particularly in primary care settings, is critical for ensuring that patients' preferences are documented and respected throughout their healthcare journey [5]. Kavalieratos et al. found that early integration of palliative care, including ACP, is associated with improved patient and caregiver outcomes, including reduced hospitalizations and enhanced quality of life [16]. This is particularly pertinent in a clinic setting where continuity of care and clear communication are crucial for effective patient management. The importance of healthcare provider training in ACP was emphasized by Lum et al., who found that well-trained providers are more likely to initiate and complete ACP discussions [17].

The importance of addressing ACP during public health crises, such as the COVID-19 pandemic, has also been highlighted in recent literature. It was noted that the pandemic has brought ACP to the forefront, emphasizing the need for healthcare providers to engage in these discussions proactively [18]. This aligns with the findings from this study, which underscore the necessity of standardized ACP practices to prepare for unforeseen circumstances and ensure that patient's wishes are respected even during crises [18]. A study by Curtis et al. during the pandemic also highlighted the critical role of ACP in reducing the burden on healthcare systems by ensuring that patient care aligns with their preferences, particularly when resources are limited [18].

In addition to primary care and public health settings, ACP has been shown to have significant implications in specialty care. For instance, Mack et al. demonstrated that end-of-life discussions in oncology settings are associated with better patient outcomes and reduced distress [19]. Similarly, Mullick et al. highlighted the importance of integrating ACP into routine practice across various specialties to ensure that patients' wishes are consistently respected [20]. Studies in cardiology and nephrology settings, such as those by Schell et al., have further validated the positive impact of ACP on patient satisfaction and care quality [21].

This project on improving ACP discussions demonstrates a significant advancement in patient care practices [1]. The standardized approach not only educates and empowers patients but also provides a replicable model for other healthcare institutions [5]. The findings, supported by recent literature, underscore the importance of targeted ACP interventions and their positive impact on patient outcomes and healthcare practices [9]. Future research should explore the long-term effects of such interventions on healthcare utilization and patient satisfaction, particularly in diverse and underserved populations. Moreover, the ongoing development of ACP tools and resources, as discussed by Bristowe et al., will be essential in supporting both patients and healthcare providers in the future [22].

Limitations

This study has several limitations that should be acknowledged. First, the sample size was relatively small, with only 52 patients completing the pre-intervention survey and 23 patients completing the post-intervention survey. This small sample size may limit the generalizability of the findings to the broader population served by the internal medicine clinic. Larger studies with more diverse patient populations are needed to confirm these results and determine whether the observed improvements in ACP discussions can be replicated in different settings.

Second, the study was conducted over a short duration of only three months. This limited timeframe may not have been sufficient to capture the long-term effects of the intervention on patient behavior and outcomes. For instance, while the post-intervention survey indicated an increased likelihood of patients completing advanced directives, it remains unclear whether these intentions translated into actual behavior change over time. A longer follow-up period would be necessary to assess the sustainability of the intervention's impact.

Finally, the study relied on self-reported data from patients, which may be subject to response bias. Patients may have provided socially desirable answers, particularly regarding their comfort with end-of-life discussions and their intentions to complete advanced directives. Additionally, the study did not include a control group, making it difficult to determine whether the observed changes were solely attributable to the intervention or if other external factors may have influenced the results. Future studies should consider including a control group and using objective measures, such as the actual completion rates of advanced directives, to strengthen the validity of the findings.

## Conclusions

This project on improving ACP discussions demonstrates a significant advancement in patient care practices. The standardized approach not only educates and empowers patients but also provides a replicable model for other healthcare institutions. The findings, supported by recent literature, underscore the importance of targeted ACP interventions and their positive impact on patient outcomes and healthcare practices.

## Figures and Tables

**Figure 6:**
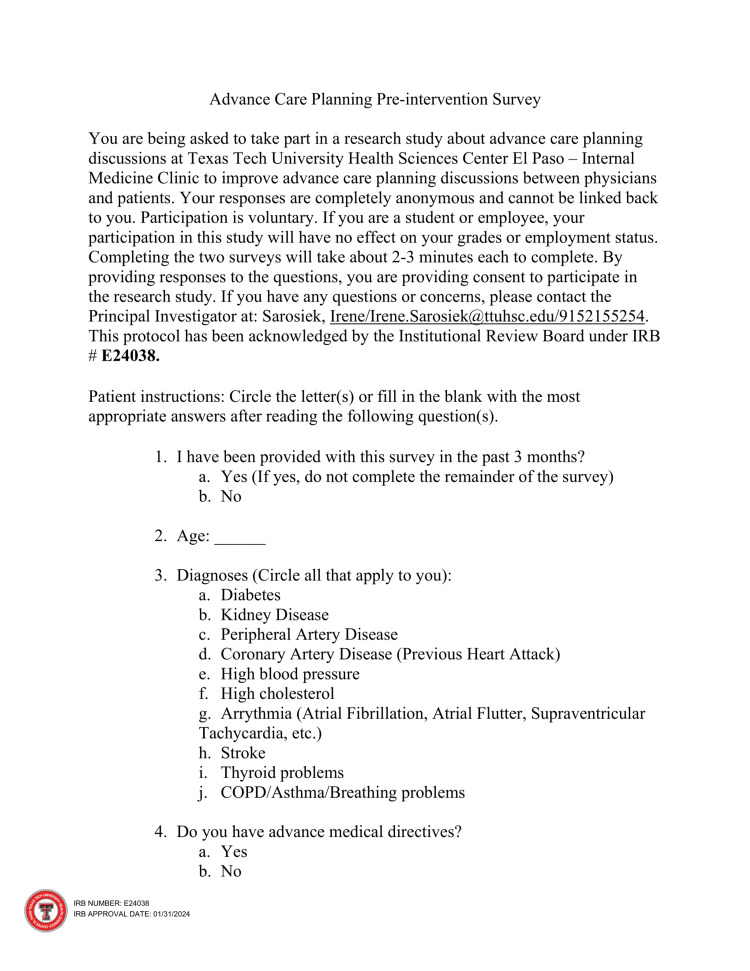
Advance care planning pre-intervention survey English part 1 of 2

**Figure 7:**
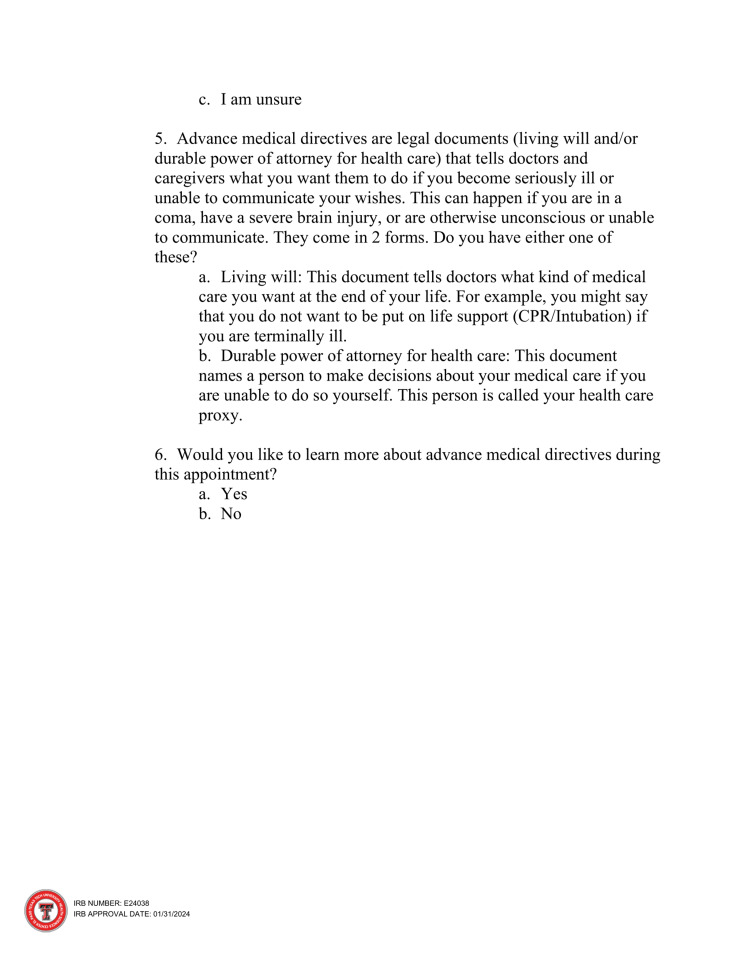
Advance care planning pre-intervention survey English part 2 of 2

**Figure 8:**
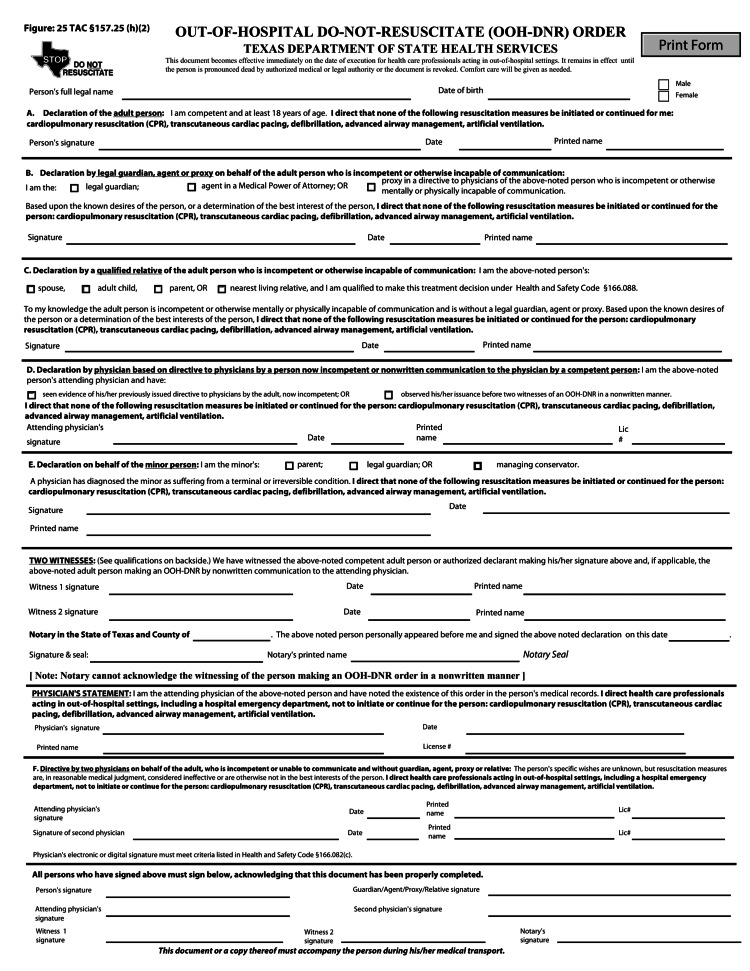
Out-of-hospital do-not-resuscitate (OOH-DNR) form English part 1 of 2

**Figure 9:**
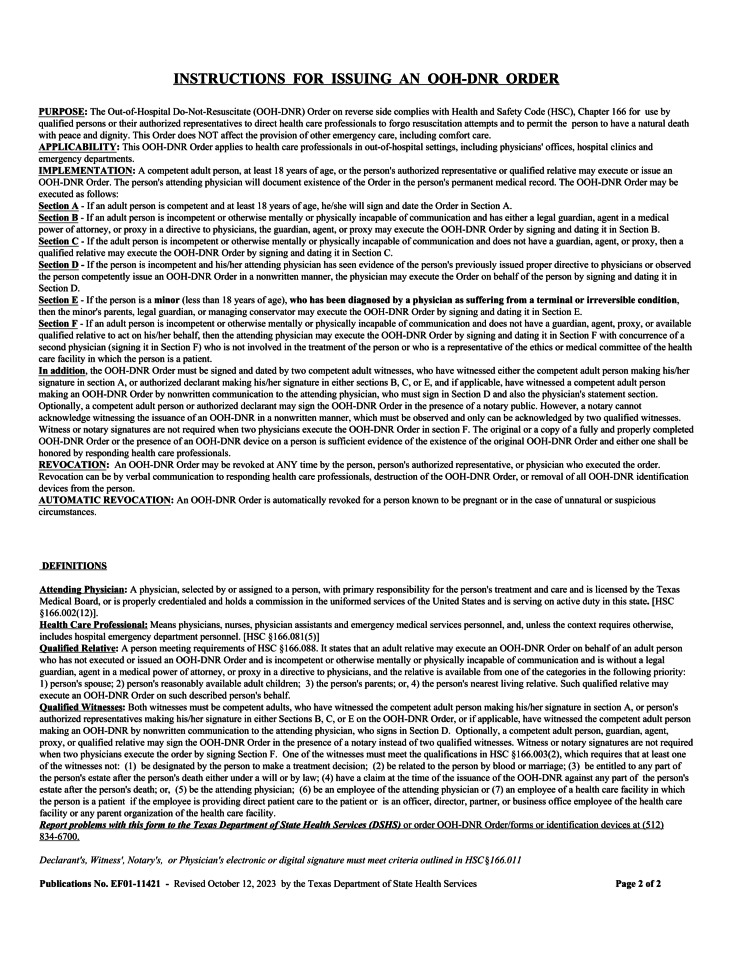
Out-of-hospital do-not-resuscitate (OOH-DNR) form English part 2 of 2

**Figure 10:**
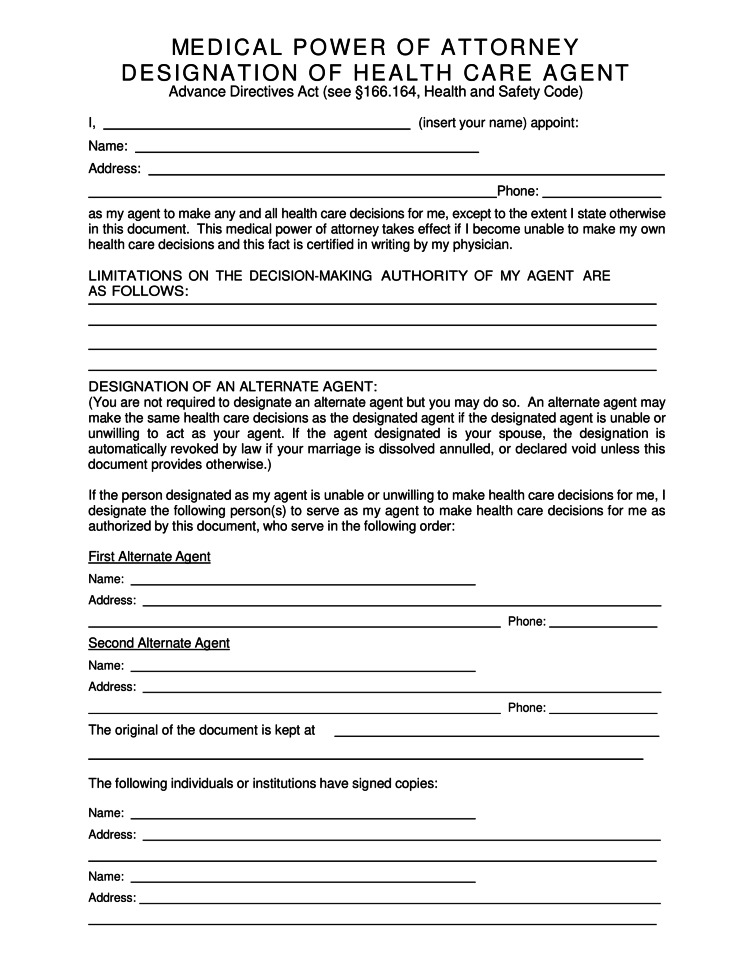
Medical power of attorney form English part 1 of 4

**Figure 11:**
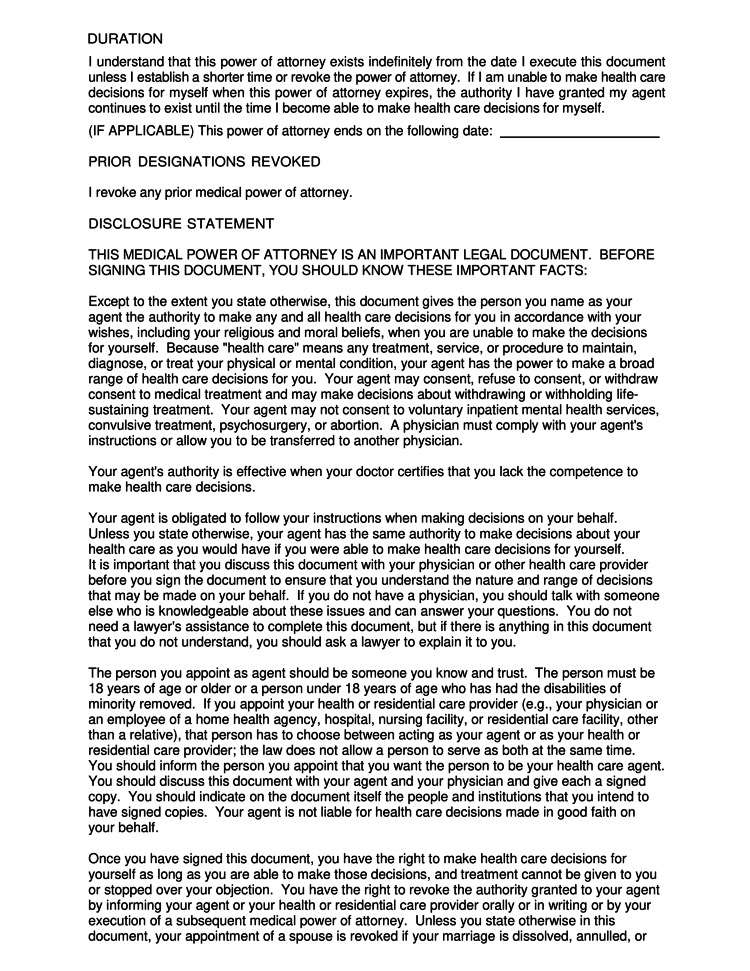
Medical power of attorney form English part 2 of 4

**Figure 12:**
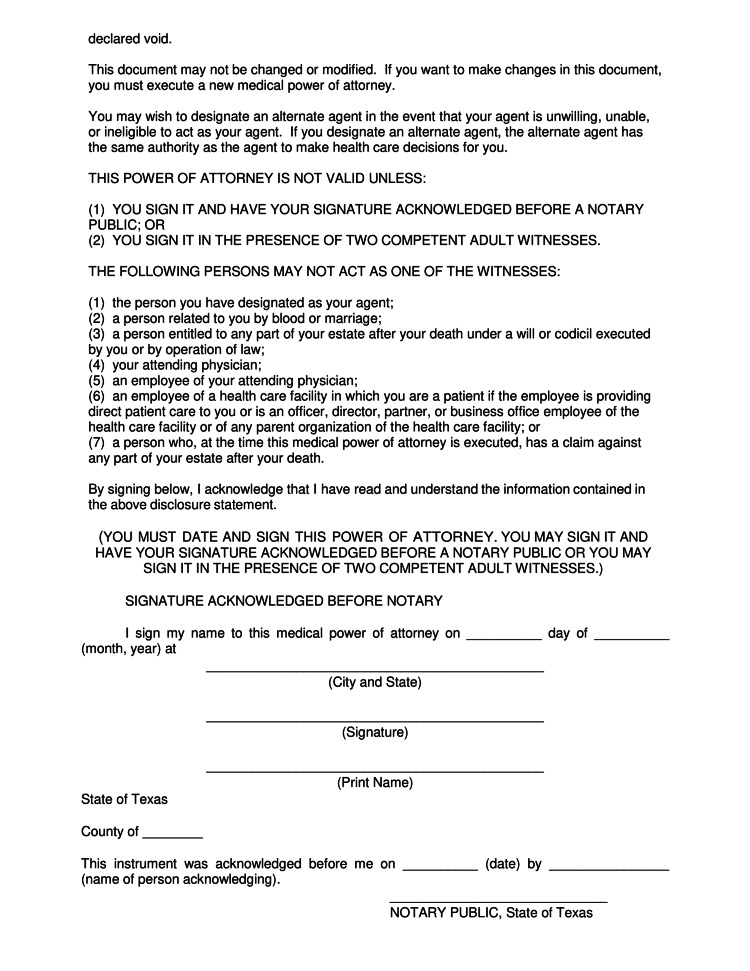
Medical power of attorney form English part 3 of 4

**Figure 13:**
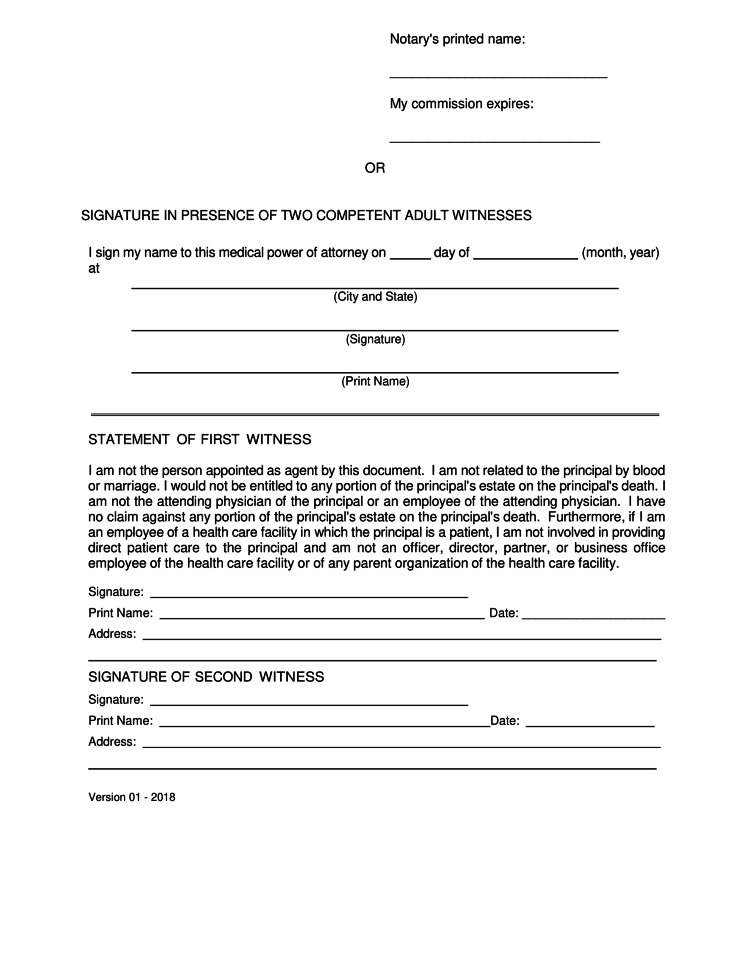
Medical power of attorney form English part 4 of 4

**Figure 14:**
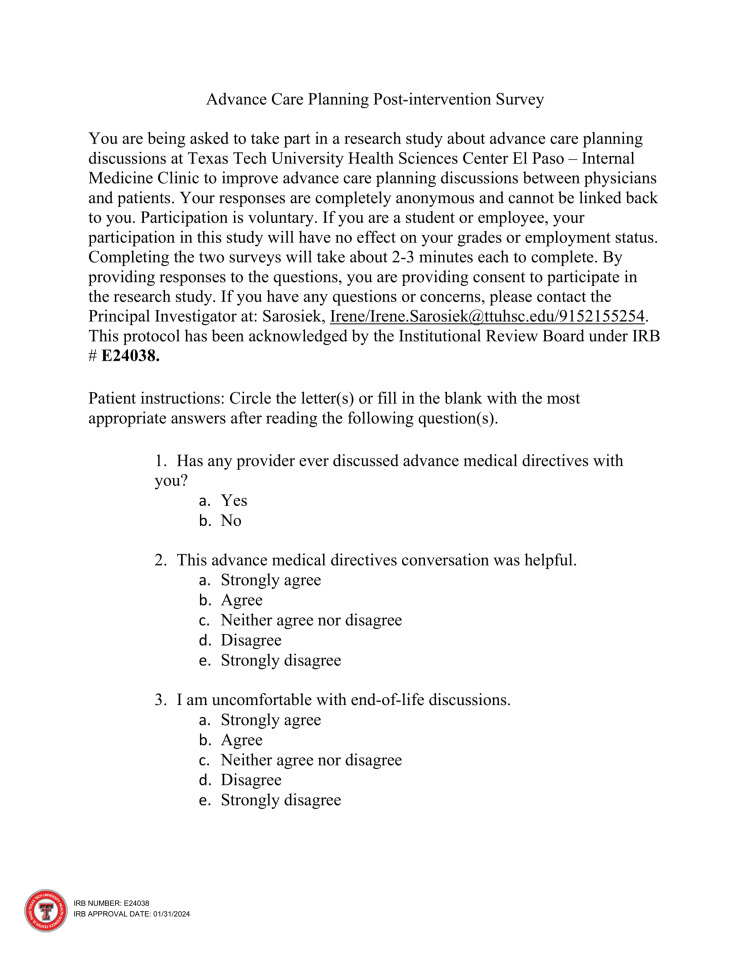
Advance care planning post-intervention survey English part 1 of 2

**Figure 15:**
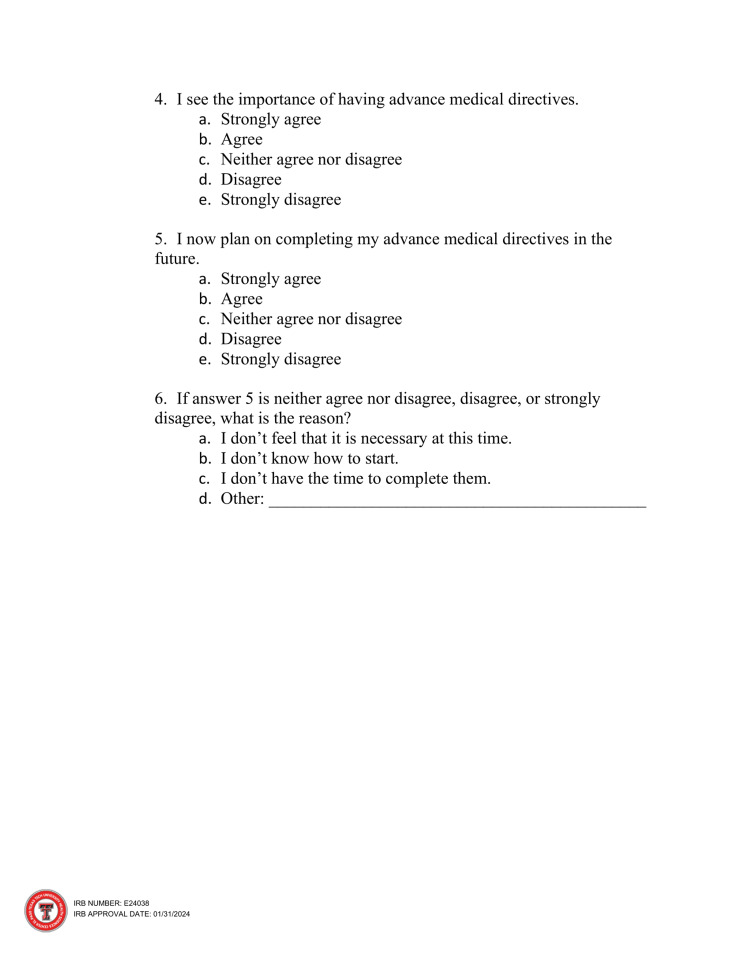
Advance care planning post-intervention survey English part 2 of 2

## Appendices

Appendix A

Appendix B 

Appendix C 

Appendix D 
